# Trial of Tetrachloride of Carbon as an Anæsthetic—Dangerous Effects

**Published:** 1867-12

**Authors:** E. Andrews

**Affiliations:** Professor of Principles and Practice of Surgery, Chicago Medical College; 81 Monroe St., Chicago


ARTICLE L.

### TRIAL OF TETRACHLORIDE OF CARBON AS AN ANÆSTHETIC.—DANGEROUS EFFECTS.

By E. ANDREWS, M.D., Prof. of Principles and Practice of Surgery, Chicago Medical College.

In a letter written a few months ago, to the EXAMINER, I called attention to the new anæsthetic called tetrachloride of carbon, introduced by Dr. PROTHEROE SMITH, of London. Dr. SMITH had used the article in about one hundred cases; and was disposed to believe it safer than chloroform and far more agreeable than ether. On my return to this country, I brought a sample of it with me, from the same establishment which supplies it to Dr. SMITH. I had a patient, upon whom it became necessary to perform the operation of resection of the hip-joint, and who had previously suffered so much nausea after the inhalation of ether, that he very much disliked to take it a second time. As one of the chief advantages of the tetrachloride of carbon is its freedom from nauseating effects, I deemed it best to use it in this case. Having no such inhaler as is used by

Dr. SMITH, I employed a napkin, placed in a paper cone, and held a short distance from the face, as in giving chloroform. My friend, Dr. SHERMAN, whose experience in giving anæsthetics amounts to some thousands of cases, took charge of the inhalation, and proceeded with rather more caution than he would with chloroform. Nothing remarkable occurred at first, but after the lapse of a few minutes, the assistant, whose duty it was to watch the pulse, observed that it increased suddenly in frequency, so that in a short time he was unable to count it. At the same time, the patient, who was not yet unconscious, complained of a violent pain, as of cramp, in the vicinity of the heart, and after a moment more, the pulse and respiration both suddenly ceased. The patient's head was spasmodically drawn backward, the countenance looked pale and deathly, and the pupils of the eyes dilated until the iris could scarcely be seen. Artificial respiration was at once commenced, and strong aqua ammoniæ was rubbed in the nostrils, under which treatment, the patient revived again, although to all appearance almost dead. The anæsthesia was then completed by concentrated sulphuric ether, without further accident, and the carious bone excised in the usual manner. I do not think that there remained any prolonged unfavorable effect after the use of the tetrachloride, but the sudden advent of such urgent and dangerous symptoms made a strongly unfavorable impression on my mind, for the patient was much nearer death than I ever saw one go under ether. I certainly shall not venture on the use of the article again, unless very extensive experience by others demonstrates its safety.

It is proper to state that the patient was in a very exhausted and anæmic condition from the effects of disease, and was operated on as a last, desperate resort, having no other hope of life. He rallied from the operation pretty well, without showing any signs of injury from the tetrachloride, but died, subsequently, from exhaustion.

*81 Monroe St., Chicago, Nov. 4, 1867.*

