# Correction

**Published:** 1868-01

**Authors:** 


### CORRECTION.

Editor of Examiner.—In the article on "Tetrachloride of Carbon," of last month, there is an error, implying that Dr. Sherman's experience in giving anæsthetics amounts to "some thousands of cases." It should read "above a thousand of cases." E. ANDREWS, M.D.

